# Socioeconomic Variation in the Prevalence, Introduction, Retention, and Removal of Smoke-Free Policies among Smokers: Findings from the International Tobacco Control (ITC) Four Country Survey

**DOI:** 10.3390/ijerph8020411

**Published:** 2011-01-31

**Authors:** Brian A. King, Andrew J. Hyland, Ron Borland, Ann McNeill, K. Michael Cummings

**Affiliations:** 1 Department of Health Behavior, Roswell Park Cancer Institute, Elm and Carlton Streets, Buffalo, NY 14263, USA; E-Mails: brian.alexander.king@gmail.com (B.A.K.); michael.cummings@roswellpark.org (K.M.C.); 2 Vic Health Center for Tobacco Control, The Cancer Council Victoria, Carlton, VIC 3053, Australia; E-Mail: ron.borland@cancervic.org.au; 3 UK Centre for Tobacco Control Studies, Division of Epidemiology & Public Health, University of Nottingham, Nottingham NG51PB, UK; E-Mail: ann.mcneill@nottingham.ac.uk

**Keywords:** tobacco smoke pollution, smoking, public policy, socioeconomic factors, United States, Canada, Australia, United Kingdom

## Abstract

**Introduction:**

Exposure to secondhand smoke causes premature death and disease in non-smokers and indoor smoke-free policies have become increasingly prevalent worldwide. Although socioeconomic disparities have been documented in tobacco use and cessation, the association between socioeconomic status (SES) and smoke-free policies is less well studied.

**Methods:**

Data were obtained from the 2006 and 2007 Waves of the International Tobacco Control Four Country Survey (ITC-4), a prospective study of nationally representative samples of smokers in Canada, the United States, the United Kingdom, and Australia. Telephone interviews were administered to 8,245 current and former adult smokers from October 2006 to February 2007. Between September 2007 and February 2008, 5,866 respondents were re-interviewed. Self-reported education and annual household income were used to create SES tertiles. Outcomes included the presence, introduction, and removal of smoke-free policies in homes, worksites, bars, and restaurants.

**Results:**

Smokers with high SES had increased odds of both having [OR: 1.54, 95% CI: 1.27–2.87] and introducing [OR: 1.49, 95% CI: 1.04–2.13] a total ban on smoking in the home compared to low SES smokers. Continuing smokers with high SES also had decreased odds of removing a total ban [OR: 0.44, 95% CI: 0.26–0.73]. No consistent association was observed between SES and the presence or introduction of bans in worksites, bars, or restaurants.

**Conclusions:**

The presence, introduction, and retention of smoke-free homes increases with increasing SES, but no consistent socioeconomic variation exists in the presence or introduction of total smoking bans in worksites, bars, or restaurants. Opportunities exist to reduce SES disparities in smoke-free homes, while the lack of socioeconomic differences in public workplace, bar, and restaurant smoke-free policies suggest these measures are now equitably distributed in these four countries.

## 1. Introduction

Exposure to secondhand smoke (SHS) causes significant morbidity and mortality among both adults and children who do not smoke [1,2]. SHS is responsible for an estimated 600,000 premature deaths per year worldwide, 31% of which occur among children [3]. Accordingly, Article 8 of the World Health Organization Framework Convention on Tobacco Control requires ratifying nations to pass measures that provide universal protection from tobacco smoke in indoor environments, including public places, worksites, and public transport [3].

Global estimates indicate that one-third of adults are exposed to SHS on a regular basis and approximately 700 million children (40%) are exposed in the home [3]. Fortunately, restrictions on smoking in indoor public places are becoming increasingly normative in many countries [4,5]. The implementation of such policies significantly reduces SHS exposure [6] and can have an immediate and sustained impact on smoking-related health outcomes, including the reduced incidence of heart attacks in the general population [7]. There is also evidence to suggest that public smoking restrictions stimulate the adoption of voluntary smoke-free policies in the home [8], both of which have been shown to have a beneficial impact on smoking behavior change [9–11].

Socioeconomic status (SES) is strongly associated with smoking related knowledge, consumption, and cessation [12–15] and socioeconomic disparities in smoking prevalence have continued to widen over time in many Western countries [16–18]. Substantial socioeconomic differences in adult mortality have also been linked to the effects of smoking [19–21]. Commonly cited hypotheses for the underlying processes that produce these social inequalities include variations in educational attainment, propensity and self-efficacy toward quitting, and deprivation [22]. In contrast, few studies have assessed the association between SES and population-level strategies to reduce tobacco use and SHS exposure, such as the implementation of indoor smoking restrictions. The limited number of population-based studies of smoke-free worksites indicate that overall exposure and policy non-compliance are disproportionately higher among individuals with lower SES [23–27]. Similarly, assessments of smoking restrictions in homes have found smoke-free policy prevalence is positively associated with increasing SES [8,26,28]. Socioeconomic disparities have also been observed in receptivity toward such policies, with individuals of lower SES being less likely to support restrictions in indoor public places and worksites [29]. There is also evidence to suggest that the benefits of smoke-free policies may be irrespective of socioeconomic status; one recent study found that comprehensive policies prohibiting smoking in all worksites, restaurants, and bars are independently associated with reduced smoking participation and consumption, regardless of education and income [30].

Given the magnitude and persistence of the association between SES and tobacco use, the reduction of social inequalities in smoking-related indicators has become an important public health priority for many national governments and policymakers [31]. A key strategy used to achieve this goal has been the implementation and intensification of smoking restrictions in indoor environments [3]. However, there has been limited research on the extent to which these restrictions vary among socioeconomic groups both across and within countries. More specifically, it is of particular importance to determine whether the recent worldwide proliferation of smoke-free policies [4,5] has been uniform across socioeconomic groups.

The primary objective of this study was to utilize population data from Canada, the United States (US), the United Kingdom (UK), and Australia to assess socioeconomic and national variations in the prevalence, introduction, retention, and removal of smoke-free policies in various indoor environments, including homes, worksites, bars, and restaurants. The assessment of policy change was of particular interest in the UK, where national legislation prohibiting smoking in worksites, bars, and restaurants was implemented among most respondents between data collection Waves. A secondary objective was to identify sociodemographic predictors of these policy-related indicators by environment type. Understanding sociodemographic disparities in access to smoke-free environments may help mitigate such disparities in tobacco use.

## 2. Methods

This study reports data from Waves 5 and 6 of the International Tobacco Control Four Country Survey (ITC-4), a prospective cohort study to monitor the impact of national level tobacco control policies in four countries: Canada, the US, the UK, and Australia. The ITC-4 was initiated in 2002 with over 2,000 adult smokers recruited by probability sampling methods in each of the four countries. In subsequent follow-up surveys of the cohort, recruited samples are replenished after attrition to ensure a sample size of at least 2,000 per country at each Wave. A detailed description of the design features, data collection methods, and analytic strategies of the ITC-4 study have been previously reported elsewhere [32].

Participants were identified using stratified random digit dialing and interviews were conducted using computer assisted telephone interview (CATI) software by multiple research firms. The interviews were conducted in either English or French (francophone areas of Canada), but strict protocols were followed to ensure methodological congruity across firms and between the two languages [32]. The study protocol received ethical review and clearance from the Institutional Review Boards or Research Ethics Boards at the University of Waterloo (Canada), Roswell Park Cancer Institute (USA), the University of Illinois at Chicago (USA), the University of Stirling (UK), the University of Nottingham (UK), and the Cancer Council Victoria (Australia).

### 2.1. Participants

Selected participants included 8,245 current and former adult smokers who were interviewed as part of Wave 5 of the ITC-4 survey between October 2006 and February 2007 (Canada, n = 2,023; the US, n = 2,034; the UK, n = 2,019; and Australia, n = 2,169). Between September 2007 and February 2008, a total of 5,866 of these participants (71.1%) were successfully re-interviewed in Wave 6 (Canada, n = 1,459, 72.1%; the US, n = 1,291, 63.5%; the UK, n = 1,484, 73.5%; and Australia, n = 1,632, 75.2%). In addition, another 2,329 individuals were recruited as part of the Wave 6 replenishment sample (Canada, n = 556; the US, n = 711; the UK, n = 523; and Australia, n = 539).

### 2.2. Measures

#### 2.2.1. Socioeconomic status

Questions related to respondent education and annual household income were used to create previously utilized indices of self-reported socioeconomic status [14,15]. Respondents were categorized into three levels of educational attainment: low (less than high school diploma), moderate (high school diploma), and high (some university training or a university degree). Similarly, respondents from the US, Canada, and Australia were categorized into three levels of annual household income: low ($29,999 or less), moderate ($30,000 to $59,999), and high ($60,000 or more). In an effort to account for variation in currency values, respondents from the UK were categorized into annual household income categories using a modified rubric: low (£15,000 or less), moderate (£15,001 to £30,000), and high (£30,001 or more). These education and income categories were then combined to create a previously utilized composite measure for SES [33] as follows: low (low education and low income), moderate (low income and moderate education, low income and high education, moderate income and low education, and high income and low education), and high (moderate income and moderate education, moderate income and high education, high income and moderate education, and high income and high education). Respondents who did not report either income (5.7%) or education (0.3%) were excluded from the analysis. The aforementioned categorization methods resulted in a similar distribution across countries and tertile divisions.

#### 2.2.2. Smoke-Free policy presence, introduction, retention, and removal

The presence of a smoke-free home policy was assessed using the question, ‘Which of the following best describes smoking in your home?’, with the following response options: ‘smoking is allowed anywhere in your home’, ‘smoking is never allowed anywhere in your home’, or ‘something in between’. A respondent was considered to be covered by a total ban in their home if they answered ‘smoking is never allowed anywhere in your home’ and a partial ban if they answered ‘something in between’.

The presence of a smoke-free bar policy was assessed using the question, ‘Which of the following best describes the rules about smoking in drinking establishments, bars, and pubs where you live?’, with the following response options: ‘smoking is not allowed in any indoor area’, ‘smoking is allowed in some indoor areas’, or ‘no rule or restrictions’. A respondent was considered to be covered by a total ban in local bars if they answered ‘smoking is not allowed in any indoor area’ and a partial ban if they answered ‘smoking is allowed in some indoor areas’.

The presence of a smoke-free restaurant policy was assessed using the question, ‘Which of the following best describes the rules about smoking in restaurants and cafes where you live?’, with the following response options: ‘smoking is not allowed in any indoor area’, ‘smoking is allowed in some indoor areas’, ‘smoking is allowed in all indoor areas’, or ‘every restaurant, café has its own rules’. A respondent was considered to be covered by a total ban in local restaurants if they answered ‘smoking is not allowed in any indoor area’ and a partial ban if they answered ‘smoking is allowed in some indoor areas’.

The presence of a smoke-free worksite policy was assessed among individuals employed outside the home using the question, ‘Which of the following best describes the smoking policy where you work?’, with the following response options: ‘smoking is not allowed in any indoor area’, ‘smoking is allowed only in some indoor areas’, or ‘smoking is allowed in any indoor areas’. A respondent was considered to be covered by a total ban in their worksite if they answered ‘smoking is not allowed in any indoor area’ and a partial ban if they answered ‘smoking is allowed only in some indoor areas’.

The presence of a smoke-free home, bar, restaurant, or worksite policy was defined as being covered by a total ban at Wave 5, whereas the introduction of a smoke-free policy was defined as being covered by no ban or a partial ban at Wave 5 and a total ban at Wave 6. Retention of a smoke-free policy was defined as being covered by a total ban at both Waves, while retention of smoking was defined as being covered by either no ban or a partial ban at both Waves. Given the non-voluntary nature of smoking restrictions in public areas, removal of a smoke-free policy was only assessed among those with smoke-free homes and was defined as being covered by a total ban at Wave 5 and either no ban or a partial ban at Wave 6 [8,34].

#### 2.2.3. Smoking status

Smoking status was determined at Wave 5 using answers to questions related to respondents’ daily, weekly, or monthly smoking rates and whether they had quit smoking between study recruitment and the Wave 5 survey [32]. At Wave 6, respondents were asked whether they were still smoking or not, and whether any quit attempts were made to arrive at their current smoking or quitting status. A ‘current smoker’ was defined as any respondent who reported smoking daily, weekly, or monthly at the time of survey. In contrast, a ‘former smoker’ was defined as any respondent who either remained quit since the time of last survey Wave completion or who made an attempt to stop smoking since the time of last survey Wave completion and was also quit for a month or more at the time of current survey Wave.

#### 2.2.4. Covariates

Covariates included: age at Wave 5 (18–24, 25–39, 40–54, or 55+ years), gender (male or female), minority status (mainstream/non-identified minority or identified minority), country of residence (Canada, US, UK, or Australia), children less than 18 years old in the household (yes or no), non-smoking adults in the household (yes or no), Heaviness of Smoking Index (HSI) (range from 0 to 6) [35], smoke-free home policy (partial/no ban or total ban), smoke-free bar policy (partial/no ban or total ban), smoke-free restaurant policy (partial/no ban or total ban), and smoke-free worksite policy (partial/no ban, total ban, or not employed outside the home). Support for smoking bans in worksites, bars, and restaurants were also assessed using a five level index in which a value of ‘1’ represented the belief that smoking should be allowed in all four of the aforementioned venues types, a value of ‘5’ represented the belief that smoking should be prohibited in all four venue types, and intermediate values representing the belief that smoking should be prohibited in one, two, or three of the venue types.

### 2.3. Data Analysis

Data analyses were conducted using SPSS version 14.0 (SPSS Inc., Chicago, IL). All bivariate analyses were restricted to participants with complete data for the indicator of interest and stratified by both participant smoking status and country of residence. Cross-sectional data were weighted by age, gender, and region [32] to provide representative population estimates for the following four outcomes: total smoking ban in the home, worksite, bars, or restaurants. Bivariate analyses pertaining to cohort data were unweighted and included the following four outcomes: introduction of a smoke-free policy, retention of no total ban, removal of total ban, or retention of a smoke-free policy. For each of the aforementioned bivariate analyses, a chi-square test was used to determine statistically significant differences across subgroups (α = 0.05). A binary logistic regression model was also constructed to identify significant predictors of policy prevalence (total ban vs. partial/no ban at Wave 5), introduction (introduction vs. no introduction at Wave 6 among those with no ban at Wave 5), and removal (removal *vs.* no removal at Wave 6 among those with a total ban at Wave 5) among current and continuing smokers, while simultaneously adjusting for the effects of the following covariates: SES, age, gender, minority status, country of residence, children less than 18 years of age in the household, non-smoking adults in the household, HSI, attitudes toward smoke-free policies in public places, and the presence of current total smoking bans in local bars, restaurants, and worksites. To verify whether the effects of SES were consistent across countries, a separate model was also constructed that included the aforementioned covariates and a statistical interaction term for SES and country. Due to limited sample size, it was not possible to construct any regression models among former smokers.

## 3. Results

### 3.1. Smoke-Free Home Policies

Table 1 presents the prevalence, introduction, retention, and removal of smoke-free home policies by SES and country of residence. Overall, the proportion of current smokers who reported that smoking was never allowed anywhere in their home (total ban) was greatest among respondents from Australia (Wave 5: 46.4%; Wave 6: 50.1%) and lowest among those from the UK (Wave 5: 23.9%; Wave 6: 29.4%) at both Waves. Among former smokers, respondents from Australia had the highest proportion of total bans at both Waves (Wave 5: 69.4%; Wave 6: 74.9%), while those from the UK (53.4%) and Canada (60.9%) had the lowest proportions in Waves 5 and 6, respectively. Following stratification by SES, the proportion of current smokers with a total ban increased with increasing SES at both Waves, regardless of country of residence. A similar trend was observed across SES levels among former smokers in Canada, while a significant lack of trend was observed in Australia. With the exception of the US and Australia, both the introduction and retention of a total ban in the home among continuing smokers also increased with increasing SES.

Table 2 presents the findings of a binary logistic regression analysis to identify predictors of the presence, introduction, and removal of a total ban on smoking in the home. Among current and continuing smokers from all four countries combined, those with either ‘moderate’ [Odds Ratio (OR): 1.31, 95% Confidence Interval (CI): 1.10–1.55] or ‘high’ [OR: 1.54, 95% CI: 1.27–2.87] SES had an increased odds of having a total ban in Wave 5, as well as a decreased odds of removal of a ban by Wave 6 [‘Moderate’ OR: 0.52, 95% CI: 0.34–0.80; ‘High’ OR: 0.44, 95% CI: 0.26–0.73]. Additional predictors of increased odds of a policy in Wave 5 were male gender, the presence of children in the home, the presence of an adult non-smoker in the home, greater support for smoking bans in public venues, and residence in the US or Australia. Predictors of increased odds of removal of a total ban were higher Heaviness of Smoking Index (HSI) and residence in the UK, while predictors of decreased odds included greater support for smoking bans in public venues, presence of an adult non-smoker in the home, and residence in Australia. Among continuing smokers with no total ban in Wave 5, those with ‘high’ SES had an increased odds of adopting a total ban by Wave 6 [OR: 1.49, 95% CI: 1.04–2.13]. Additional predictors of increased odds of total ban introduction were greater support for smoking bans in public venues, the presence of a non-smoking adult in the home, and residence in the UK or Australia, while predictors of decreased odds were older age and higher HSI. There was no significant interaction observed between SES and country in any of the regression models, and thus the findings are not presented here.

### 3.2. Smoke-Free Bar Policies

Table 3 presents the prevalence and introduction of smoke-free bar policies by SES and country of residence. Overall, the proportion of both current and former smokers who reported that smoking was not allowed in any indoor area of local bars (total ban) was greatest among respondents from Canada in Wave 5 (current: 83.6%; former: 83.0%) and those from the UK, where a national ban on smoking in indoor public places was implemented between Waves, in Wave 6 (current: 97.1%; former: 95.3%). Between Waves 5 and 6, relative increases of 79.7% and 50.6% were observed in the proportion of current smokers with a total ban in the UK and Australia, respectively. Similar increases were also observed among former smokers in these two countries (UK: 81.1%; Australia: 45.3%). Following stratification by SES, no consistent association was observed across countries with regard to SES and either the presence or introduction of a total smoking ban in bars.

Table 2 presents the findings of a binary logistic regression analysis to identify predictors of the presence and introduction of total bans on smoking in local bars. There were no SES differences observed; however, predictors of increased odds of a total ban in Wave 5 were older age, not being employed outside the home, the presence of a total ban in restaurants, and greater support for smoking bans in public venues. In contrast, predictors of decreased odds of a total ban in Wave 5 were residence in the US, UK, or Australia. Predictors of increased odds of introducing a total ban between Waves were the presence of a total ban in restaurants, greater support for smoking bans in public venues, and residence in either the UK or Australia, while a predictor of decreased odds was residence in the US. There was no significant interaction observed between SES and country in either regression model, and thus the findings are not presented here.

### 3.3. Smoke-Free Restaurant Policies

Table 4 presents the prevalence and introduction of smoke-free restaurant policies by country of residence. Overall, the proportion of both current and former smokers who reported that smoking was not allowed in any indoor area of local restaurants (total ban) was greatest among respondents from Canada in Wave 5 (current: 91.5%; former: 92.7%) and the UK, where a national ban on smoking in indoor public places was implemented between Waves, in Wave 6 (current: 97.1%; former: 98.2%). In contrast, the proportion of respondents with such a policy was lowest among those from the UK in Wave 5 (current: 27.5%; former: 32.0%) and the US in Wave 6 (current: 65.0%; former: 60.9%). Between Waves 5 and 6, relative increases of 71.7% and 67.4% were observed among current and former smokers in the UK, respectively. Following stratification by SES, no consistent association was observed across countries with regard to SES and either the presence or introduction of a total smoking ban in local restaurants.

Table 2 presents the findings of a binary logistic regression analysis to identify predictors of the presence and introduction of total bans on smoking in local restaurants. No SES differences were observed with respect to smoke-free restaurant policy implementation. Among all current smokers, predictors of increased odds of a total ban in Wave 5 were older age, the presence of a total ban in bars, greater support for smoking bans in public venues, and having either a total ban in the worksite or not being employed outside of the home. In contrast, predictors of decreased odds were minority status and residence in either the US or UK. Predictors of increased odds of introducing a total ban between Waves among continuing smokers were greater support for smoking bans in public venues and residence in the UK, while a predictor of decreased odds was residence in the US. There was no significant interaction observed between SES and country in either regression model, and thus the findings are not presented here.

### 3.4. Smoke-Free Worksite Policies

Table 5 presents the prevalence and introduction of smoke-free worksite policies by country of residence among those employed for wages at the time of interview. Overall, the proportion of current smokers who reported that smoking was not allowed in any indoor area of their worksite (total ban) was greatest among respondents from Canada in Wave 5 (88.2%) and those from the UK, where a national ban on smoking in indoor public areas was implemented between Waves, in Wave 6 (96.1%). The US had the lowest proportion at both Waves (Wave 5: 76.8%; Wave 6: 75.9%). Among former smokers, the proportion of respondents with such a policy in Wave 5 was the greatest in the US (92.7%), but lowest in Wave 6 (83.0%). Following stratification by SES, the proportion of current smokers with a total smoking ban in the worksite increased with increasing SES in Canada and the U.S. in Wave 5, but no significant trends were apparent in Wave 6. In the UK, the proportion of former smokers with a total smoking ban in the worksite increased with increasing SES in Wave 5. Between Waves, the introduction of a total ban among continuing smokers significantly decreased with increasing SES in Canada, the U.S. and the U.K.

Table 2 presents the findings of a binary logistic regression analysis to identify predictors of the presence and introduction of total bans on smoking in the worksite. Among all current smokers combined, both those with ‘moderate’ [OR: 1.38, 95% CI: 1.06–1.82] and ‘high’ [OR: 2.23, 95% CI: 1.63–3.04] SES had an increased odds of having a total ban on smoking in the worksite in Wave 5; however, rates of smoke-free policy introduction were comparable by SES with lower point estimates among those with the highest SES compared to those with low SES. Among current smokers from all four countries combined, predictors of increased odds of a total ban in Wave 5 were the presence of a total ban in local bars or restaurants and greater support for smoking bans in public venues, while predictors of decreased odds were male gender and higher HSI. No multivariate association was observed between socioeconomic status and the introduction of a total ban in Wave 6. The only predictor of increased odds of introducing a total ban between Waves among continuing smokers was residence in the UK. There was no significant interaction observed between SES and country in either regression model, and thus the findings are not presented here.

## 4. Discussion

This study used nationally representative samples of current and former smokers from Canada, the US, the UK, and Australia to examine socioeconomic and national variations in the prevalence, introduction, retention, and removal of smoke-free policies in indoor environments, including private homes, worksites, and local bars and restaurants. The data indicate that smokers with higher SES are more likely to have, introduce, and retain total smoking bans in the home. Current smokers with higher SES were also more likely to have a total smoking ban in the workplace; however, the rate of smoke-free policy adoption in the workplace was comparable by SES group. No consistent association was observed between SES and any smoke-free policies among former smokers, or in bar and restaurants policies among current smokers. The implications of these findings are two-fold. First, the impact of recent efforts to expand the proliferation of smoke-free policies in bars and restaurants in these four countries has been seemingly uniform across those serving different socioeconomic groups; although smoke-free workplaces have previously been more common in high SES occupations, this disparity appears to have disappeared. On balance, the evidence indicates that smoke-free policies in public places are not being implemented differentially by the socioeconomic status of smokers.

Among continuing smokers, multivariate analyses indicate a clear and consistent relationship between higher SES and both the presence and introduction of smoking bans in the home. Given the potentially central role that smokers have in determining the smoke-free status of their home, this suggests some reluctance by lower SES smokers to be subjected to home smoking bans. This finding may be a consequence of having a higher likelihood of smokers in their social circle, limited access to outdoor smoking areas, and/or greater overall deprivation [22]. The present findings also indicate that nicotine dependence and attitudes toward smoke-free public environments are strong predictors of policy presence, introduction, and removal among continuing smokers, irrespective of SES. These findings are consistent with that of Borland *et al.* [8] and provide an evidence base for ongoing and future efforts to promote smoke-free homes, which have previously been shown to substantially reduce secondhand smoke exposure [2] and to have a beneficial impact on smoking behavior [9–11]. More specifically, strategies to enhance smoke-free home adoption should concentrate on the provision of cessation services and initiatives to further denormalize tobacco. The implementation of such strategies would be particularly beneficial in the UK, which had the lowest proportion of smoke-free home prevalence and retention; however, this finding was not surprising considering that the UK has traditionally lagged behind Canada, the US, and Australia in smoke-free policy implementation and support [8,34,36]. Nonetheless, it is important to note that the UK had the highest rate of smoke-free home policy introduction among continuing smokers between Waves.

While this study used parallel methods to explore predictors of smoke-free policies in various environments, care needs to be taken in interpreting the results. Although smokers are likely to have a significant role in determining the smoke-free status of their home, the same is not true for public environments. Smokers can choose to avoid environments with restrictions they dislike; therefore, sociodemographic predictors may reflect selective choice of venue for worksites, restaurants, and bars rather than, or as well as, the propensity of proprietors to differentially impose bans based upon their clientele. Nonetheless, the finding that SES is not such a factor among smokers is reassuring.

No consistent relationship was observed across countries with regard to SES and the introduction of smoke-free worksites. Continuing smokers with higher SES were at increased odds of having a total ban on smoking in the worksite at Wave 5, which may be a consequence of the propensity of lower SES individuals to work blue collar or service professions in environments that have not been traditionally covered by smoke-free policies [37]. However, this disparity appeared to attenuate by Wave 6. The rate of introduction of smoke-free worksite policies was comparable by SES, and the point estimates, while not statistically significant, indicate that introduction may now be greater in lower SES smokers. These findings suggest that worksites are now catching up in the adoption of smoke-free policies.

National variations in smoke-free policy introduction were also observed. More specifically, respondents in the UK had increased odds of reporting the introduction of bar, restaurant, and worksite policies between Waves. This finding can be attributed to the comprehensive national smoke-free legislation that was implemented between survey Waves in three of the four countries that comprise the UK; the fourth country, Scotland, had previously implemented such legislation prior to Wave 5 [38]. This increase in policy introduction is encouraging, as it confirms that most UK smokers are both aware of, and seemingly compliant with, the new smoking restrictions in these environments. Increased odds of smoke-free bar introduction was also observed in Australia, where several states, including the most populated state of New South Wales, either implemented or strengthened smoke-free policies in such establishments between Waves. In addition, an increased odds of smoke-free home introduction was observed in both the UK and Australia. This increase may be partly a result of the aforementioned public smoke-free policy implementation, which has previously been shown to be an independent predictor of smoke-free home adoption [8].

Limitations to the study include: (1) the data were obtained using respondent-reported measures of smoke-free policy presence. However, previous studies of indoor smoke-free policies and SHS have found that respondent reports are significantly correlated with established ordinances and biological measures, thereby confirming the validity of respondent-reported indicators [34,39]; and (2) respondents with missing education or income data were excluded from the analysis. However, the proportion of respondents with missing education or income data was less than 6%.

In conclusion, the findings of this study demonstrate that the presence, introduction, and retention of smoke-free homes are greater among individuals with higher SES, but no consistent socioeconomic variation exists in the presence or introduction of total smoking bans in worksites, bars, or restaurants. This indicates that opportunities exist to reduce socioeconomic disparities in smoke-free homes, while the lack of SES differences in public workplace, bar, and restaurant policies suggests these measures are now equitably distributed in these four countries.

## Figures and Tables

**Table 1.** Prevalence and introduction of smoke-free homes by socioeconomic status and country.

**Variables**	**Socioeconomic Status**															
	**Canada**				**United States**				**United Kingdom^b^**				**Australia**			
	**Low**	**Mod.**	**High**	**All**	**Low**	**Mod.**	**High**	**All**	**Low**	**Mod.**	**High**	**All**	**Low**	**Mod.**	**High**	**All**
Wave 5 ^a^																
Current Smokers																
Total ban	21.2	30.9	42.9	32.6	24.0	33.8	50.5	36.0	14.5	26.5	27.4	23.9	34.1	49.6	49.1	46.4
Partial	29.5	31.9	33.3	31.9	31.1	28.8	23.9	28.0	50.7	47.7	51.5	49.3	31.8	29.5	31.4	30.4
None	49.3	37.2	23.8	35.5	44.9	37.4	25.6	36.0	34.8	25.8	21.1	26.8	34.1	20.9	19.5	23.2
n	298	845	468	1,611 *	346	911	430	1,687 *	406	809	323	1,538*	369	950	360	1,679 *
Former Smokers																
Total ban	43.5	57.1	69.2	60.6	52.6	62.0	74.5	65.5	51.8	51.0	58.8	53.4	66.6	73.3	63.2	69.4
Partial	21.7	31.3	24.3	27.8	26.3	25.0	18.1	22.4	32.1	37.3	35.0	35.6	16.7	20.3	32.1	23.4
None	34.8	11.6	6.5	11.6	21.1	13.0	7.4	12.1	16.1	11.7	6.2	11.0	16.7	6.4	4.7	7.2
n	26	138	99	263 *	46	105	82	233	64	147	75	286	50	191	104	345 *

Wave 6 ^a^																
Current Smokers																
Total ban	21.2	29.5	45.0	35.7	24.3	29.2	46.2	35.5	17.5	32.4	32.9	29.4	36.8	52.5	53.3	50.1
Partial	36.9	34.7	28.5	32.1	31.9	30.5	28.0	29.7	45.7	37.7	42.8	41.2	30.9	28.9	28.7	29.2
None	41.9	35.8	26.5	32.2	43.8	40.3	25.8	34.8	36.8	29.9	24.3	29.4	32.3	18.6	18.0	20.7
n	255	603	721	1,579 *	332	635	674	1,641 *	366	634	466	1,466 *	341	811	529	1,681 *
Former Smokers																
Total ban	47.4	56.3	68.3	60.9	60.0	67.0	74.5	70.6	63.3	64.0	67.2	65.0	74.0	72.9	77.2	74.9
Partial	31.6	30.3	21.4	26.2	32.0	18.2	20.7	20.9	24.0	25.4	24.8	24.9	13.0	18.3	18.5	17.7
None	21.0	13.4	10.3	12.9	8.0	14.8	4.8	8.5	12.7	10.6	8.0	10.1	13.0	8.8	4.3	7.4
n	40	104	141	285	31	89	124	244	86	135	112	333	50	155	154	359
	
Between Waves among Continuing Smokers																
Introduce smoke-free	4.7	5.9	10.7	7.1	7.7	8.4	4.9	7.3	9.6	10.8	13.8	11.1	9.7	8.9	13.3	10.0
Retain smoke-free	13.5	23.4	36.2	25.7	15.5	23.3	35.6	25.1	6.2	16.4	20.2	14.6	27.8	40.1	42.5	37.8
Retain smoking	75.4	66.1	47.8	62.1	71.1	63.8	54.9	62.9	78.1	66.2	62.8	68.6	55.6	46.5	38.8	46.9
Remove smoke-free	6.4	4.6	5.3	5.1	5.7	4.5	4.6	4.7	6.1	6.6	3.2	5.7	6.9	4.5	5.4	5.3
n	171	563	318	1,052*	194	514	164	972 *	260	518	218	996 *	259	628	240	1,127 *

a Percentages are based on weighted data.

b A national policy prohibiting smoking in all worksites, restaurants, and bars was implemented between Waves 5 and 6 United Kingdom. The fourth country, Scotland, implemented such a policy prior to Wave 5.

* Statistically significant variation across groups (χ ^2^, p < 0.05).

**Table 2.** Predictors of presence, introduction, and removal of smoke-free policies among smokers, binary logistic regression.

Predictors			**Odds Ratio [95% Confidence Interval]**						
	**Homes**			**Bars**		**Restaurant**		**Worksites ^d^**	

	**Presence^a^** (n = 5,991)	**Introduction ^b^** (n = 2,634)	**Removal ^c^** (n = 1,216)	**Presence ^a^** (n = 5,991)	**Introduction ^b^** (n = 1,926)	**Presence ^a^** (n = 5,991)	**Introduction ^b^** (n = 1,244)	**Presence ^a^** (n = 3,807)	**Introduction ^b^** (n = 344)
								
Socioeconomic Status									
Low	1.00	1.00	1.00	1.00	1.00	1.00	1.00	1.00	1.00
Moderate	**1.31 [1.10–1.55]**	1.06 [0.77–1.46]	**0.52 [0.34–0.80]**	0.93 [0.77–1.10]	1.14 [0.81–1.60]	1.18 [0.97–1.44]	0.94 [0.61–1.45]	**1.38 [1.06–1.82]**	0.48 [0.22–1.03]
High	**1.54 [1.27–2.87]**	**1.49 [1.04–2.13]**	**0.44 [0.26–0.73]**	0.93 [0.75–1.14]	1.06 [0.71–1.57]	1.21 [0.96–1.54]	0.98 [0.59–1.63]	**2.23 [1.63–3.04]**	0.55 [0.23–1.34]

Age (years)									
18–24	1.00	1.00	1.00	1.00	1.00	1.00	1.00	1.00	1.00
25–39	1.02 [0.81–1.30]	0.65 [0.40–1.07]	**0.37 [0.20–0.69]**	1.15 [0.87–1.52]	0.73 [0.41–1.31]	1.28 [0.94–1.74]	0.82 [0.38–1.78]	1.00 [0.70–1.43]	2.16 [0.75–6.23]
40–54	**0.69 [0.55–0.87]**	**0.45 [0.28–0.73]**	0.65 [0.36–1.15]	1.31 [1.00–1.71]	0.72 [0.42–1.26]	**1.55 [1.15–2.10]**	0.81 [0.39–1.69]	1.20 [0.85–1.69]	1.47 [0.54–3.99]
55+	**0.69 [0.53–0.89]**	**0.43 [0.26–0.73]**	0.59 [0.31–1.13]	**1.43 [1.06–1.91]**	0.56 [0.31–1.02]	**1.63 [1.18–2.26]**	0.73 [0.34–1.60]	0.98 [0.66–1.47]	1.08 [0.34–3.40]

Gender									
Female	1.00	1.00	1.00	1.00	1.00	1.00	1.00	1.00	1.00
Male	**1.42 [1.25–1.61]**	1.09 [0.86–1.39]	1.23 [0.87–1.73]	0.91 [0.79–1.04]	0.96 [0.74–1.24]	1.04 [0.89–1.21]	1.01 [0.72–1.41]	**0.41 [0.34–0.50]**	0.68 [0.39–1.18]

Minority Status									
Mainstream/Non–Minority	1.00	1.00	1.00	1.00	1.00	1.00	1.00	1.00	1.00
Minority	0.96 [0.79–1.17]	1.08 [0.72–1.62]	1.56 [0.97–2.50]	1.19 [0.96–1.48]	1.17 [0.78–1.75]	**0.70 [0.55–0.89]**	1.01 [0.62–1.65]	0.87 [0.65–1.17]	0.63 [0.29–1.38]

Country									
Canada	1.00	1.00	1.00	1.00	1.00	1.00	1.00	1.00	1.00
United States	**1.44 [1.20–1.73]**	1.19 [0.80–1.76]	0.76 [0.45–1.28]	**0.24 [0.20–0.29]**	**0.55 [0.37–0.82]**	**0.17 [0.13–0.22]**	**0.24 [0.13–0.45]**	0.78 [0.57–1.06]	0.84 [0.37–1.89]
United Kingdom	**0.67 [0.54–0.83]**	**1.50 [1.02–2.21]**	**1.90 [1.08–3.33]**	**0.15 [0.12–0.19]**	**42.6 [24.8–73.3]**	**0.08 [0.06–0.10]**	**11.7 [5.74–23.9]**	0.74 [0.54–1.03]	**14.2 [5.26–38.5]**
Australia	**1.75 [1.47–2.08]**	**1.89 [1.34–2.68]**	**0.62 [0.39–0.99]**	**0.12 [0.09–0.14]**	**4.41 [2.98–6.54]**	1.02 [0.77–1.36]	1.66 [0.80–3.47]	0.78 [0.58–1.05]	2.00 [0.92–4.36]

Children in Home									
No	1.00	1.00	1.00	1.00	1.00	1.00	1.00	1.00	1.00
Yes	**2.08 [1.83–2.37]**	1.08 [0.83–1.41]	0.71 [0.49–1.01]	1.01 [0.87–1.17]	0.83 [0.62–1.10]	1.01 [0.85–1.20]	1.11 [0.76–1.61]	1..06 [0.87–1.29]	1.33 [0.76–2.33]

Non–Smoking Adults in Home	1.00	1.00	1.00	NI	NI	NI	NI	NI	NI
No	**2.17 [1.92–2.45]**	**1.51 [1.19–1.91]**	**0.55 [0.39–0.77]**						
Yes									

Smoke Free–Home Policy									
Partial/None	NI	NI	NI	1.00	1.00	1.00	1.00	1.00	1.00
Total Ban				0.96 [0.83–1.12]	0.95 [0.72–1.27]	1.11 [0.93–1.32]	1.07 [0.73–1.57]	1.00 [0.81–1.24]	1.08 [0.59–1.97]

Smoke Free–Bar Policy									
Partial/None	1.00	1.00	1.00	NI	NI	1.00	1.00	1.00	1.00
Total Ban	0.95 [0.82–1.10]	0.95 [0.71–1.27]	1.05 [0.70–1.59]			**14.0 [11.7–16.8]**	1.32 [0.74–2.34]	**1.33 [1.04–1.70]**	0.61 [0.31–1.20]

Smoke–Free Restaurant									
Partial/None	1.00	1.00	1.00	1.00	1.00	NI	NI	1.00	1.00
Total Ban	1.10 [0.93–1.30]	1.03 [0.75–1.43]	1.12 [0.69–1.80]	**13.9 [11.6–16.6]**	**1.45 [1.08–1.95]**			**1.61 [1.25–2.06]**	0.78 [0.38–1.63]

Smoke–Free Worksite									
Partial/None	1.00	1.00	1.00	1.00	1.00	1.00	1.00	NI	NI
Total Ban	0.99 [0.80–1.22]	1.14 [0.75–1.74]	0.89 [0.51–1.55]	1.23 [0.97–1.57]	0.98 [0.65–1.49]	**1.58 [1.23–2.02]**	1.09 [0.67–1.77]	NI	NI
Not employed outside home	0.93 [0.74–1.17]	1.03 [0.66–1.62]	1.29 [0.72–2.30]	**1.31 [1.02–1.70]**	1.08 [0.70–1.66]	**1.46 [1.13–1.89]**	0.83 [0.51–1.37]	NI	NI

Heaviness of Smoking Index	**0.74 [0.71–0.77]**	**0.87 [0.80–0.94]**	**1.32 [1.18–1.48]**	1.01 [0.97–1.06]	1.02 [0.93–1.11]	1.01 [0.96–1.07]	1.08 [0.97–1.20]	**0.90 [0.84–0.96]**	0.87 [0.74–1.03]

Public Smoking Ban Support	**1.34 [1.27–1.42]**	**1.24 [1.12–1.38]**	**0.85 [0.73–0.98]**	**1.33 [1.26–1.41]**	**1.20 [1.06–1.35]**	**1.32 [1.23–1.41]**	**1.22 [1.04–1.42]**	**1.59 [1.46–1.73]**	1.23 [0.96–1.57]

Note: NI= Not included in model. Statistically significant odds ratios are noted in bold.

a Presence of total smoking ban among current smokers at Wave 5.

b Implementation of a total smoking ban between Wave 5 and Wave 6 among continuing smokers.

c Removal of total smoking ban between Wave 5 and Wave 6 among continuing smokers.

d Among individuals employed for wages outside the home.

**Table 3.** Prevalence and introduction of smoke-free bars by socioeconomic status and country.

**Variables**	**Socioeconomic Status**															
	**Canada**				**United States**				**United Kingdom ^b^**				**Australia**			
	**Low**	**Mod.**	**High**	**All**	**Low**	**Mod.**	**High**	**All**	**Low**	**Mod.**	**High**	**All**	**Low**	**Mod.**	**High**	**All**
Wave 5 ^a^																
Current Smokers																
Total ban	82.7	82.3	86.4	83.6	37.6	36.9	44.9	39.1	27.7	18.4	15.5	19.7	41.2	44.9	41.6	43.4
Partial	15.2	15.3	12.3	14.4	44.2	47.9	42.9	45.9	51.7	57.3	65.9	58.2	53.9	52.1	56.4	53.4
None	2.1	2.4	1.3	2.0	18.2	15.2	12.2	15.0	20.6	24.3	18.6	22.1	4.9	3.0	2.0	3.2
n	293	823	462	1,578	311	853	416	1,580 *	365	785	314	1,464 *	350	916	344	1,610

Former Smokers																
Total ban	87.0	87.0	76.6	83.0	54.3	46.3	59.1	52.9	20.0	13.5	25.0	18.0	50.0	48.7	46.1	48.1
Partial	8.7	12.3	23.4	16.3	20.0	34.7	31.2	30.9	64.0	66.7	59.2	64.0	50.0	48.7	52.0	49.8
None	4.3	0.7	0.0	0.7	25.7	19.0	9.7	16.2	16.0	19.8	15.8	18.0	0.0	2.6	1.9	2.1
n	25	136	99	260 *	42	101	81	224	58	142	73	273	46	188	99	333

Wave 6 ^a^																
Current Smokers																
Total ban	85.3	86.2	90.3	88.0	51.2	53.2	50.9	51.9	95.1	97.8	97.6	97.1	88.7	86.3	89.5	87.8
Partial	12.7	13.6	9.1	11.3	38.3	31.8	39.5	36.3	3.1	1.7	2.1	2.2	10.4	13.0	10.0	11.5
None	2.0	0.2	0.6	0.7	10.5	15.0	9.6	11.8	1.8	0.5	0.3	0.7	0.9	0.7	0.5	0.7
n	251	596	713	1,560 *	297	595	649	1,541	356	627	463	1,446	324	786	521	1,631
Former Smokers																
Total ban	87.5	88.8	91.5	90.0	47.6	49.3	51.8	50.5	91.3	96.2	97.0	95.3	83.7	83.6	93.9	87.9
Partial	12.5	11.2	7.7	9.7	47.6	38.4	40.0	40.2	7.2	2.3	3.0	3.7	16.3	13.9	6.1	10.9
None	0.0	0.0	0.8	0.3	4.8	12.3	8.2	9.3	1.5	1.5	0.0	1.0	0.0	2.5	0.0	1.2
n	39	100	141	280	28	85	116	229	85	132	111	328	44	150	152	346 *

Between Waves among Continuing Smokers																
Introduce smoke-free	7.8	8.2	4.5	7.1	14.5	16.9	14.9	15.9	64.3	78.1	79.3	74.9	42.2	40.9	48.2	42.8
Retain smoke-free	79.5	81.4	83.9	81.8	36.1	34.6	38.1	35.9	30.3	19.5	17.3	21.7	46.0	46.5	41.1	45.2
Retain smoking	7.2	7.5	10.0	8.2	45.2	45.7	43.5	44.9	4.6	1.8	3.4	2.9	10.5	11.4	9.8	10.9
Remove smoke-free	5.5	2.9	1.6	2.9	4.2	2.8	3.5	3.3	0.8	0.6	0.0	0.5	1.3	1.2	0.9	1.1
n	166	547	310	1,023	166	462	255	883	238	497	208	943 *	239	596	224	1,059

a Percentages are based on weighted data.

b A national policy prohibiting smoking in all worksites, restaurants, and bars was implemented between Waves 5 and 6 United Kingdom. The fourth country, Scotland, implemented such a policy prior to Wave 5.

* Statistically significant variation across groups (χ^2^, p < 0.05).

**Table 4.** Prevalence and introduction of smoke-free restaurants by socioeconomic status and country.

**Variables**	**Socioeconomic Status**															
	**Canada**				**United States**				**United Kingdom^b^**				**Australia**			
	**Low**	**Mod.**	**High**	**All**	**Low**	**Mod.**	**High**	**All**	**Low**	**Mod.**	**High**	**All**	**Low**	**Mod.**	**High**	**All**
Wave 5 ^a^																
Current Smokers																
Total ban	90.8	90.0	94.6	91.5	50.9	53.2	58.8	54.2	34.3	26.4	24.0	27.5	81.5	86.3	82.5	84.5
Partial	6.7	7.0	4.6	6.3	27.8	26.8	24.5	26.4	33.4	36.2	36.8	35.7	9.1	7.3	12.2	8.8
None	2.5	3.0	0.8	2.2	21.3	20.0	16.7	19.4	32.3	37.4	39.2	36.8	9.4	6.4	6.3	6.7
n	294	842	467	1,603	337	906	429	1,672	380	800	320	1,500*	363	940	358	1,661

Former Smokers																
Total ban	96.2	91.2	93.9	92.7	60.5	56.0	64.2	60.1	36.4	33.3	26.3	32.0	85.7	92.1	86.7	89.7
Partial	0.0	6.1	5.6	5.4	23.6	25.0	22.3	23.7	30.4	25.9	39.5	30.5	11.6	4.5	8.6	6.6
None	3.8	2.7	0.5	1.9	15.9	19.0	13.5	16.2	33.2	40.8	34.2	37.5	2.7	3.4	4.7	3.7
n	26	137	99	262	46	105	82	233	63	144	73	280	49	192	103	344

Wave 6 ^a^																
Current Smokers																
Total ban	96.0	93.8	95.7	95.0	69.9	64.1	63.8	65.0	95.2	97.5	97.8	97.1	90.7	94.0	94.7	93.7
Partial	2.3	4.6	3.5	3.7	16.1	18.4	17.7	17.7	2.3	1.7	1.9	1.9	2.9	3.0	2.8	2.9
None	1.7	1.6	0.8	1.3	14.0	17.5	18.5	17.3	2.5	0.8	0.3	1.0	6.4	3.0	2.5	3.4
n	254	604	721	1,579	323	628	674	1,625	360	632	466	1,458*	332	802	527	1,661

Former Smokers																
Total ban	91.9	93.3	95.9	94.4	58.3	60.7	61.4	60.9	95.9	100	97.3	98.2	93.3	94.0	97.5	95.5
Partial	0.0	5.8	4.1	4.3	28.0	15.5	20.7	19.7	1.4	0.0	1.8	0.9	4.4	2.4	1.9	2.4
None	8.1	0.9	0.0	2.3	13.7	23.8	17.9	19.4	2.7	0.0	0.9	0.9	2.3	3.6	0.6	2.1
n	40	104	141	285	31	88	122	241	87	134	112	333	48	153	154	355

Between Waves among Continuing Smokers																
Introduce smoke-free	3.5	4.4	2.2	3.6	18.5	14.9	14.0	15.3	57.2	69.0	69.0	66.0	11.0	9.8	13.9	11.0
Retain smoke-free	92.9	91.5	93.4	92.3	47.8	48.7	51.9	49.4	38.7	28.1	27.8	30.7	81.8	84.8	83.6	83.9
Retain smoking	1.8	2.1	1.6	1.9	31.5	32.1	31.1	31.7	2.9	2.5	2.3	2.6	3.6	2.6	0.8	2.4
Remove smoke-free	1.8	2.0	2.8	2.2	2.2	4.3	3.0	3.6	1.2	0.4	0.9	0.7	3.6	2.8	1.7	2.7
n	169	563	317	1,049	184	505	264	953	243	513	216	972	253	620	238	1,111

a Percentages are based on weighted data.

b A national policy prohibiting smoking in all worksites, restaurants, and bars was implemented between Waves 5 and 6 in three United Kingdom. The fourth country, Scotland, implemented such a policy prior to Wave 5.

* Statistically significant variation across groups (χ^2^, p < 0.05).

**Table 5.** Prevalence and introduction of smoke-free worksites by socioeconomic status and country.

**Variables**	**Socioeconomic Status**															
	**Canada**				**United States**				**United Kingdom ^b^**				**Australia**			
	**Low**	**Mod.**	**High**	**All**	**Low**	**Mod.**	**High**	**All**	**Low**	**Mod.**	**High**	**All**	**Low**	**Mod.**	**High**	**All**
Wave 5 ^a^																
Current Smokers																
Total ban	85.8	86.4	91.8	88.2	67.5	75.7	83.2	76.8	74.2	75.8	80.3	77.0	88.0	83.7	91.6	86.3
Partial	12.0	9.8	7.2	9.1	21.2	17.4	13.4	16.7	16.5	19.1	16.4	18.0	11.1	12.6	4.5	10.2
None	2.2	3.8	1.0	2.7	11.3	6.9	3.4	6.5	9.3	5.1	3.3	5.0	0.9	3.7	3.9	3.5
n	131	566	373	1,070 *	130	500	298	928*	108	532	256	896	123	646	293	1,062 *
Former Smokers																
Total ban	100	84.8	91.9	88.5	82.4	87.7	98.7	92.7	68.2	77.6	90.0	80.9	93.3	90.6	94.7	92.2
Partial	0.0	6.7	7.0	6.5	5.9	3.5	0.0	2.0	18.2	18.7	7.1	14.6	6.7	5.6	3.2	4.8
None	0.0	8.5	1.1	5.0	11.7	8.8	1.3	5.3	13.6	3.7	2.9	4.5	0.0	3.8	2.1	3.0
n	10	96	78	184	13	53	62	128	22	100	65	187*	19	149	90	258

Wave 6 ^a^																
Current Smokers																
Total ban	88.6	85.1	87.0	86.4	73.9	72.4	79.3	75.9	94.9	95.4	97.4	96.1	84.5	84.9	89.0	86.5
Partial	10.0	8.9	11.1	10.1	18.5	19.4	16.9	18.1	5.1	4.3	1.5	3.3	8.5	9.1	5.2	7.5
None	1.4	6.0	1.9	3.5	7.6	8.2	3.8	6.0	0.0	0.3	1.1	0.6	7.0	6.0	5.8	6.0
n	71	271	320	662	94	235	313	642	70	310	235	615	77	358	295	730
Former Smokers																
Total ban	90.0	86.8	84.8	86.4	100	71.4	92.6	83.0	66.7	97.5	89.6	90.0	100	83.7	89.2	87.5
Partial	10.0	13.2	6.1	9.9	0.0	21.4	7.4	13.6	25.0	0.0	10.4	8.0	0.0	9.3	5.4	6.7
None	0.0	0.0	9.1	3.7	0.0	7.2	0.0	3.4	8.3	2.5	0.0	2.0	0.0	7.0	5.4	5.8
n	9	28	33	70	2	26	29	57	12	39	43	94*	5	47	40	92

Between Waves among Continuing Smokers																
Introduce smoke-free	16.7	8.1	4.6	8.1	25.0	12.6	12.5	14.8	50.0	36.2	29.8	35.8	21.1	14.3	10.2	14.0
Retain smoke-free	83.3	73.8	80.5	77.0	43.8	61.5	70.0	60.8	40.0	61.1	64.3	59.9	71.0	70.9	79.5	73.3
Retain smoking	0.0	14.0	13.8	12.2	31.2	25.9	16.2	24.0	10.0	2.7	1.2	3.0	7.9	13.8	8.0	11.5
Remove smoke-free	0.0	4.1	1.1	2.7	0.0	0.0	1.3	0.4	0.0	0.0	4.7	1.3	0.0	1.0	2.3	1.2
n	36	172	87	295 *	48	135	80	263 *	30	185	84	299 *	38	196	88	322

a Percentages are based on weighted data.

b A national policy prohibiting smoking in all worksites, restaurants, and bars was implemented between Waves 5 and 6 in three United Kingdom. The fourth country, Scotland, implemented such a policy prior to Wave 5.

* Statistically significant variation across groups (χ^2^, p < 0.05).
